# Syncytiotrophoblast‐derived extracellular vesicles carry apolipoprotein‐E and affect lipid synthesis of liver cells in vitro

**DOI:** 10.1111/jcmm.17056

**Published:** 2021-12-10

**Authors:** Chiara Tersigni, Muhammad Furqan Bari, Shijei Cai, Wei Zhang, Neva Kandzija, Alice Buchan, Fabrizio Miranda, Nicoletta Di Simone, Christopher W. Redman, Claire Bastie, Manu Vatish

**Affiliations:** ^1^ Nuffield Department of Women’s & Reproductive Health University of Oxford Oxford UK; ^2^ Dipartimento di Scienze della Salute della Donna Fondazione Policlinico Universitario A. Gemelli IRCCS del Bambino e di Sanità Pubblica Rome Italy; ^3^ Dow Medical College Karachi Pakistan; ^4^ Radcliffe Department of Medicine University of Oxford Oxford UK; ^5^ Ovarian Cancer Cell Laboratory Weatherall Institute of Molecular Medicine University of Oxford Oxford UK; ^6^ Department of Biomedical Sciences Humanitas University Milan Italy; ^7^ IRCCS Humanitas Research Hospital Milan Italy; ^8^ Division of Biomedical Sciences Warwick Medical School University of Warwick Coventry UK

**Keywords:** apolipoprotein‐E, extracellular vesicles, lipid metabolism, liver, placenta, pregnancy

## Abstract

In normal pregnancy, hepatic metabolism adaptation occurs with an increase in lipid biosynthesis. Placental shedding of syncytiotrophoblast‐derived extracellular vesicles (STBEVs) into the maternal circulation constitutes a major signalling mechanism between foetus and mother. We investigated whether STBEVs from normal pregnant women might target liver cells in vitro and induce changes in lipid synthesis. This study was performed at the Nuffield Department of Women's & Reproductive Health, Oxford, UK. STBEVs were obtained by dual‐lobe placental perfusion from 11 normal pregnancies at term. Medium/large and small STBEVs were collected by ultracentrifugation at 10,000g and 150,000g, respectively. STBEVs were analysed by Western blot analysis and flow cytometry for co‐expression of apolipoprotein‐E (apoE) and placental alkaline phosphatase (PLAP). The uptake of STBEVs by liver cells and the effect on lipid metabolism was evaluated using a hepatocarcinoma cell line (HepG2 cells). Data were analysed by one‐way ANOVA and Student's *t* test. We demonstrated that: (a) STBEVs carry apoE; (b) HepG2 cells take up STBEVs through an apoE‐LDL receptor interaction; (c) STBEV incorporation into HepG2 cells resulted in (i) increased cholesterol release (ELISA); (ii) increased expression of the genes SQLE and FDPS (microarray) involved in cholesterol biosynthesis; (iii) downregulation of the CLOCK gene (microarray and PCR), involved in the circadian negative control of lipid synthesis in liver cells. In conclusion, the placenta may orchestrate the metabolic adaptation of the maternal liver through release of apoE‐positive STBEVs, by increasing lipid synthesis in a circadian‐independent fashion, meeting the nutritional needs of the growing foetus.

## 1 INTRODUCTION

Extracellular vesicles (EV) are produced by most cells and organs including the human placenta. During pregnancy, EVs are shed from the syncytiotrophoblast (called STBEVs), the placental layer in direct contact with maternal blood, into the maternal circulation.^1^ The release of STBEVs increases throughout gestation (and is estimated to be in excess of 3g every 24 hours at term.^2^ STBEVs are heterogeneous and include small EVs (~100 nm in diameter), medium/large EVs (0.1‐1 μm) and apoptotic bodies (0.5–5 μm)—which are a terminal cellular events and not the focus of this work. According to their biogenesis, STBEV can be separated into exosomes and microvesicles that are released constitutively or after activation in relation to pathologies, such as cancer, cardiovascular disease or cellular stress. EVs have signalling functions and the ability to dock with other cells^3^ to which they can carry and donate cargo,^4^ to be taken up and used by the recipient cell. The cargoes include regulatory molecules such as miRNAs, lipids and DNA. Uniquely, STBEVs carry placental alkaline phosphatase (PLAP), which allows identification of STBEVs as being of placental origin.^5^

Circulating extracellular vesicles are, therefore, a form of complex systemic signalling. Thus, syncytiotrophoblast (the placental epithelium bathed in maternal blood) releases STBEV, which extend the potential for feto‐placental communication in ways that have not been clearly undefined.

During pregnancy, levels of circulating triglycerides and cholesterol rise progressively with gestational age, due to the increase of maternal hepatic biosynthesis and peripheral lipolysis, and revert to normal levels postpartum.^6^, ^7^ The mechanisms by which the feto‐placental unit delivers its message of increasing energy requirements to maternal tissues are still matter of investigation.

A proteomic analysis of STBEVs obtained by dual‐lobe placental perfusion of normal term placentae revealed the presence of apolipoprotein‐E (ApoE) as a protein carried on them (Tannetta et al. unpublished). ApoE is a 299‐residue protein carried by circulating lipoproteins, mainly chylomicrons and Intermediate Density Lipoproteins (IDL).^8^ It is a key regulator of plasma lipid levels, promoting clearance of triglyceride‐rich lipoproteins through its lipid‐binding ability. In particular, the interaction between ApoE and Low‐Density Lipoproteins Receptor (LDLR) in hepatocytes mediates endocytosis of lipoprotein remnant particles and lipolysis.^8^ Since ApoE has a central role in mediating lipoprotein uptake by liver cells, we hypothesized that the expression of ApoE on STBEVs might have a role in mediating uptake of STBEVs by maternal liver cells in pregnancy, inducing metabolic changes.

## 2 MATERIALS AND METHODS

### 2.1 Human subjects

This study was approved by the Central Oxford Research Ethics Committee (07/H0607/74 and 07/H0606/148). Healthy pregnant women, undergoing elective caesarean section at term (indication for caesarean section was breech presentation or previous caesarean delivery), provided written informed consent for the use of the placental tissues and blood. Placentae were collected within 10 minutes of delivery. Normal pregnancy (NP) included healthy women with no history of hypertension or chronic illness, a singleton pregnancy without known foetal abnormality, and natural conception.

### 2.2 Tissue immunofluorescence

Placental sections were deparaffinised by successive washings in Histo‐clear 1 and 2 solutions (Dako, Glostrup, Denmark) before being rehydrated in alcohol solutions. Slides were dipped in sodium citrate buffer (10 mM, pH 6.0) (VWR, UK) for 10 minutes in a microwave oven, cooled at room temperature (R/T) and washed in PBS, before being incubated in a blocking solution (10% v/v FCS in PBS) for 1 hour. Sections were incubated overnight with 20 µg/ml of apoE primary rabbit monoclonal antibody (Abcam, Cambridge, UK) and washed before and after staining with Alexa Fluor donkey anti‐rabbit IgG secondary antibody (Life Technologies, Carlsbad, CA, USA) at 1:400 for 1 hour. Vectashield mounting medium containing DAPI (Vector Laboratories, USA) was used to counterstain the nuclei. Sections were visualized using a Leica DMIRE2 inverted fluorescence microscope. Images were taken using a Hamamatsu Orca digital camera with Simple PCI software.

### 2.3 Isolation and characterization of STBEVs

Syncytiotrophoblast‐derived extracellular vesicles were prepared using a dual‐lobe placental perfusion system and serial centrifugation protocol as we have previously described in Dragovic et al.^9^ Maternal placental perfusate was centrifuged at 10,000 × *g* to pellet the fractions enriched with the medium/large STBEVs, or microvesicles (MV), and 150,000 × *g* to pellet the small STBEVs, or nanovesicles (NV) or exosomes. Medium/large STBEVs were phenotyped using a BD LSRII flow cytometer (BD Biosciences) as described below. STBEVs’ size and concentration were further characterized using a NanoSight NS500 (Malvern, UK). Western blotting was used to confirm syncytiotrophoblast origin of STBEVs, using a specific placental marker, placental alkaline phosphatase (PLAP) and the exosomal markers (Alix, Syntenin and CD9) for the small STBEVs. PLAP is exclusively expressed in placental tissues and is commonly used to distinguish vesicles released by the placenta from those coming from other cell types. The protein concentration of STBEVs was determined by bicinchoninic acid protein assay (BCA), prior to storage at −80°C.

### 2.4 Western blotting

Immunoblotting was performed using standard protocols with plasma (1 µg), medium/large and small STBEVs (10 µg) from 11 NP women. Membranes were incubated at 4°C overnight with rabbit monoclonal antibodies against apoE or apo‐A1 (Abcam, Cambridge, UK) (1 µl/ml), or with mouse monoclonal anti‐PLAP antibody (1 µl/ml, NDOG2, in‐house antibody) in Tris‐buffered saline and 0.05% Tween 20 (TBS‐T) containing 1% BSA. After washing, membranes were then incubated with HRP‐conjugated secondary antibody (1:4000; Dako, Glostrup, Denmark) for 1 hour at R/T. Immunoblots were treated with an enhanced chemiluminescence detection system (PierceTM, Thermo Fischer Scientific, Waltham, MA. USA) and exposed to Hyperfilm ECL (GE Healthcare Life Sciences, Cleveland, Ohio, USA).

### 2.5 Immunoaffinity precipitation

According to the manufacturer's protocols, 100 µL of magnetic Dynabeads Sheep anti‐rabbit IgG (NOVEX, Life Technologies, Waltham, MA USA) were coated with 4 µg of anti‐PLAP or anti‐apoE antibodies for 30 minutes at 4°C under gentle tilting and rotation. After washing, PLAP or apoE‐coated Dynabeads were incubated with 50 µg medium/large or small STBEVs (pools of 3 patients with high expression of ApoE at Western blot analysis) for 1 hour at 4°C. After incubation, Dynabeads samples were placed in the magnet and supernatant containing PLAP or apoE‐depleted medium/large or small STBEVs were collected and stored at −20°C. To confirm apoE depletion, Western blotting was performed on apoE‐ or PLAP‐depleted STBEVs samples while 10 µg medium/large or small STBEVs not depleted for ApoE was used as a control. The whole experiment was repeated three times.

### 2.6 Nanoparticle tracking analysis

Syncytiotrophoblast‐derived extracellular vesicles diameter and concentration were measured using a NanoSight NS500 (Malvern Instruments, Malvern, UK) equipped with an sCMOS camera and the nanoparticle tracking analysis software version 2.3, Build 0033 (Malvern Instruments, Malvern, UK). The instrument was calibrated prior to our measurements, using silica 100 nm microspheres (Polysciences, Warrington, UK). Size distribution profiles and concentration of STBEVs were measured using a protocol previously described.^9^

### 2.7 Cell cultures

Human hepatocellular carcinoma human cell line (HepG2, 85011430, Sigma‐Aldrich, UK) was cultured in Dulbecco's modified Eagle's medium/Ham's F12 (DMEM/F12, Sigma‐Aldrich, UK) supplemented with 2 mM 1‐glutamine, 100 IU/ml penicillin, 100 μg/ml streptomycin and 10% (v/v) FCS (Serum Laboratories International UK) in a humidified atmosphere containing 5% CO_2_ in air at 37°C.

### 2.8 Incubation of HepG2 with STBEVs

HepG2 cells were seeded at concentration of 2 × 10^6^ cells/ml in 6 well plates (Merck Millipore, Germany). HepG2 cells were incubated for 24 hours with 2 ml of culture medium only or 2 ml of medium containing: (a) medium/large STBEVs, (b) small STBEVs, (c) medium/large STBEVs depleted of apoE, (d) small STBEVs depleted of apoE at a concentration of 50 µg/ml. After washing, cells were treated with 250 µl ice‐cold lysis buffer (10 mM Tris HCl pH 7.4) for 1 hour. Supernatants were collected after centrifugation at 15,000 × g at 4°C for 10 min. Protein content was measured by BCA prior to storage at −80°C until use. For gene expression array or qPCR, cells were washed 3 times with PBS, collected and stored in PBS or RNA later (Qiagen, Hilden, Germany) at −20°C until use.

### 2.9 Confocal analysis

HepG2 cells were seeded at low density (1 × 10^4^/ml) in Nunc™ Lab‐Tek™ II Chamber Slide™ System (Thermo Fisher Scientific, Waltham, MA USA). Two of the eight wells with HepG2 cells were pre‐incubated for 15 minutes at 37°C with 200 µl of Recombinant Human LRPAP1 (Sino Biological inc.; concentration use 1 µM), a ligand to LDLR. 1 ml aliquots (50 µg/ml) of medium/large or small STBEVs, depleted or not of apoE, were added to wells and incubated for 1 hour. Cells were treated with 0.5 ml of fixation buffer (BioLegend, San Diego, CA, USA) and then stained with wheat germ agglutinin (WGA) Alexa Fluor^®^ 488 Conjugate solution (Invitrogen, 5 µg/ml). Cells were permeabilized using the Permabilazation wash buffer (BioLegend)and treated with a blocking solution comprising 10% (v/v) FCS in PBS for 1 hour before being incubated with unlabelled NDOG2 mouse monoclonal IgG antibody (2 µg/ml) or IgG1 isotype control antibody (2 µg/ml) overnight. After washing with PBS, cells were next stained with Alexa Fluor^®^ 647 conjugate goat anti‐mouse secondary antibody (Abcam, Cambridge, UK; 1:400) for 1 hour at R/T. Nuclei were counterstained with Vectashield mounting medium containing DAPI (Vector Laboratories, USA) and cells were observed on an inverted confocal microscope (LSA 510 META, Zeiss). Images were recorded using Zen software (Rochdale, UK).

### 2.10 Flow cytometry analysis

Analyses of medium/large STBEVs and HepG2 cells were carried out by multi‐colour flow cytometry, using a BD LSRII Flow Cytometer (BD Biosciences, Oxford, UK) equipped with a 488 nm (blue) and 633 nm (red) laser. All data were analysed using FACS DIVA software (Becton Dickinson, Oxford, UK).

#### 2.10.1 Syncytiotrophoblast‐derived extracellular vesicles

Medium/Large STBEVs were samples were diluted in filtered PBS to optimize an event rate of ~300 events/s in a final volume of 300 µl. Prior to staining, samples were incubated with 10 µl of Fc receptor blocker (Miltenyi, Woking, UK) for 10 min, at 4°C. Samples were then labelled with anti‐PLAP‐PE (Biolegend UK Ltd., Cambridge, UK) or anti‐apoE‐FITC (Abcam, Cambridge, UK) for 15 min at R/T. Isotype controls were matched to their respective antibodies according to the concentration, fluorochrome type and heavy chain. Prior to data acquisition, final volumes were adjusted to 300 µl with PBS. A total of 100,000 events were collected for each sample. EV gates were set at <1 µm using fluorescent beads. The negative gates for staining were determined using isotype control tests and set at 1%.

#### 2.10.2 Cells

1–2 × 10^6^ HepG2 cells were seeded at high density in six well plates (Merck Millipore, Germany). 2 ml of culture medium containing medium/large or small STBEVs (50 µg/ml), isolated from 3 normal placentae and pooled together, depleted or not of apoE, were added in each well in a humidified atmosphere containing 5% CO2 in air at 37°C for 1 hour. Cells were then treated with 0.5 ml of fixation buffer (BioLegend) for 20 minutes before being incubated in 1.5 ml of Accutase^®^ solution (Sigma‐Aldrich) to detach the cells. Cells were centrifuged at 350g for 3 minutes, resuspended in 1X intracellular staining permabilization wash buffer (BioLegend) and centrifuges at 350g for 10 minutes. Permeabilized HepG2 cells were resuspended in PBS and blocked with 0.2 mm filtered Fc receptor blocker (10 µL; Miltenyi) for 10 min before incubation for 15 minutes of either filtered PBS (unlabelled control), NDOG2‐PE‐conjugated antibody or its IgG1‐PE‐conjugated isotype control. For cholesterol synthesis and microarray experiments, cells were in vitro synchronized for circadian rhythm by incubation with 0.1uM dexamethasone (in the culture medium) for 15 minutes, followed by washing out with fresh medium as has been previously described.^10^

#### 2.10.3 ELISA

Cholesterol content was quantified in HepG2 cell lysates using a Total Cholesterol Colorimetric/Fluorometric Assay Kit (K603, BioVision, CA, USA), according to the manufacturer's instructions. Total cholesterol concentration was calculated by absorbance (450 nm) measurement using a FLUOstar^®^ Omega microplate reader (BMG LABTECH, Germany). Data were normalized to protein content.

### 2.11 RNA isolation and mRNA‐to‐cDNA reverse transcription

After incubating HepG2 cells for 24 hours with (a) medium only (b) medium/large STBEVs, (c) small STBEVs, (d) medium/large STBEVs depleted of apoE, (e) small STBEVs depleted of apoE at a concentration of 50 µg/ml, cells were washed with warm PBS prior to RNA extraction using the RNeasy micro kit (Qiagen, Hilden, Germany). Samples (N = 10) were then treated with DNA‐free removal kit (Thermo Fisher, Waltham, Massachusetts, USA) to remove any contaminating DNA. The RNA concentration was measured using a NanoDrop ND‐1000 Spectrophotometer (Thermo Fisher) at 260 nm absorbance, and RNA purity was assessed using the 260:280 nm absorbance ratio. Samples were diluted with RNase‐free water and adjusted to 100 ng/μL concentration. RNA samples were converted into cDNA using a High capacity RNA‐cDNA conversion kit (Applied Biosystems, Thermo Fisher).

### 2.12 Microarray analysis

Microarray analysis of HepG2 cells was performed using RNA prepared and hybridized to Illumina BeadChip microarrays, following manufacturers’ protocols. Raw data were imported into the R statistical software (http://www.R‐project.org) and processed using BioConductor packages.^11^ Ingenuity Pathway Analysis (Qiagen) was used to analyse the end‐results, according to manufacturers’ instructions. The experiment has been repeated twice.

### 2.13 Quantitative polymerase chain reaction

The nucleotide Basic Local Alignment Search Tool and Primer‐BLAST were used to design and analyse the specificity of the primer sequence for CLOCK. Primer sequences are as follows: CLOCK_F GAGAGCGAAGGAAATCT, CLOCK_R GCAGCTTTGCAGGAACAAGTA, HPRT (hypoxanthine phosphoribosyltransferase) _F GCT GGTGAAAAGGACCTCT, HPRT _R CACAGGACTAGA ACACCTGC. The expression of CLOCK along with HPRT internal control was assayed using SYBR Green Mix (Bioline Reagents Ltd, UK) in a 20 μl qPCR reaction according to the manufacturer's protocols. The samples were amplified using an ABI Prism 7000 Sequence detection system (Applied Biosystems, Foster City, USA). The experiment has been repeated twice.

### 2.14 Statistical analysis

Data were analysed using GraphPad Prism 7 (GraphPad Software, CA). Differences between 2 treatments were tested for statistical significance (*p *< 0.05) using a Student's unpaired *t*‐test. Values were expressed as Mean ±Standard Error of the Mean (SEM).

## 3 RESULTS

### 3.1 Clinical characteristics of recruited patients

Experiments were performed on 11 placentae collected from normal pregnant (NP) women. Clinical characteristics of women enrolled are shown in Table 1.

**TABLE 1:** Clinical data of human subjects (n = 11) whose placentae were used for isolation of STBEV from normal pregnancy.

Age (years)	31.6 ± 1.8
BMI (kg/m^2^)	24.8 ± 1.4
Gestation Age (weeks)	40 ± 3
Primiparous (%)	5 (45.5)
Multiparous (%)	6 (54.5)
Max. Systolic pressure (mm Hg)	122 ± 3
Max. Diastolic pressure (mm Hg)	71 ± 3
New‐born weight (g)	3,768 ± 153
Male new‐borns (%)	4 (36.4)
Female new‐borns (%)	7 (63.6)

Abbreviation: BMI, body mass index.

Data are expressed ad mean ± SD or number and percentage (%) of the total population analysed (n = 11).

### 3.2 ApoE is expressed in the syncytiotrophoblast of the placenta and on STBEVs

**FIGURE 1:**
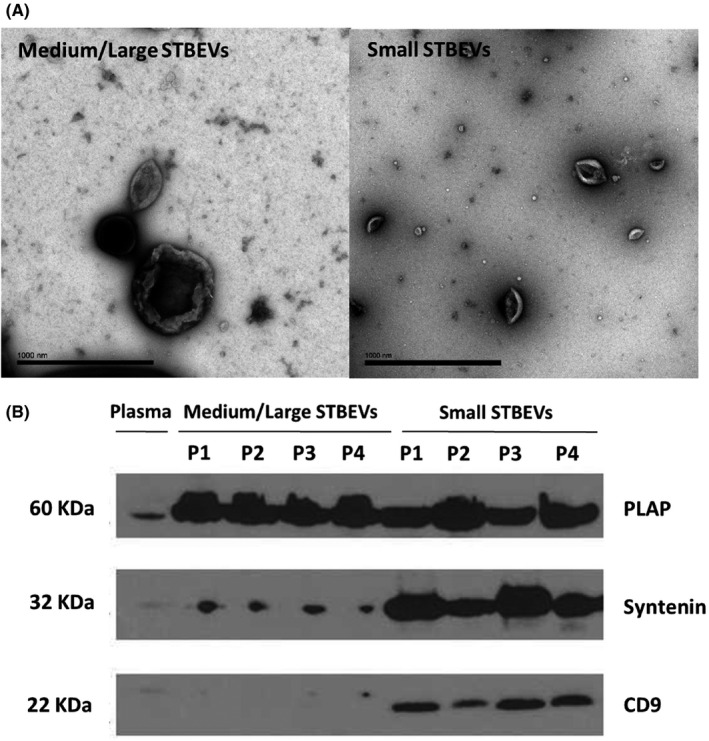
Characterisation of STBEVs derived from normal pregnancy placentae. (A) Transmission Electron Microscope (TEM) representative images of medium/large (left) STBEVs and small (right) STBEVs. Scale bars, 1000 nm. (B) Representative immunoblotting confirming placental phenotype by expression of placental alkaline phosphatase (PLAP; 60 KDa), and exosomal markers Syntenin (32 KDa) and CD9 (22 KDa) on placental lysate, medium/large and small STBEVs derived from normal pregnancy placentae. P:placental lysate. STBEVs: syncytiotrophoblast‐derived extracellular vesicles

STBEVs derived from NP placentae were characterised prior to downstream experiments by transmission electron microscopy (Figure 1A). Medium/large STBEVs (Figure 1A left) revealed a characteristic heterogeneous morphology and size consistent with previously observed findings,^5^ while small STBEVs (Figure 1A Right) were more uniformed, smaller in size and with the characteristic cup shape associated with exosomes as previously reported.^5^ Western blotting confirmed the expression of PLAP on placental lysates, with increased expression in medium/large STBEV and small STBEV confirming the previous findings.^5^ Additionally, small STBEVs were enriched for the known exosomal markers syntenin and CD9, as previously reported.^5^

Having confirmed that STBEVs that we had isolated had the correct size, morphology and vesicular markers, we went on to investigate apoE expression.

**FIGURE 2:**
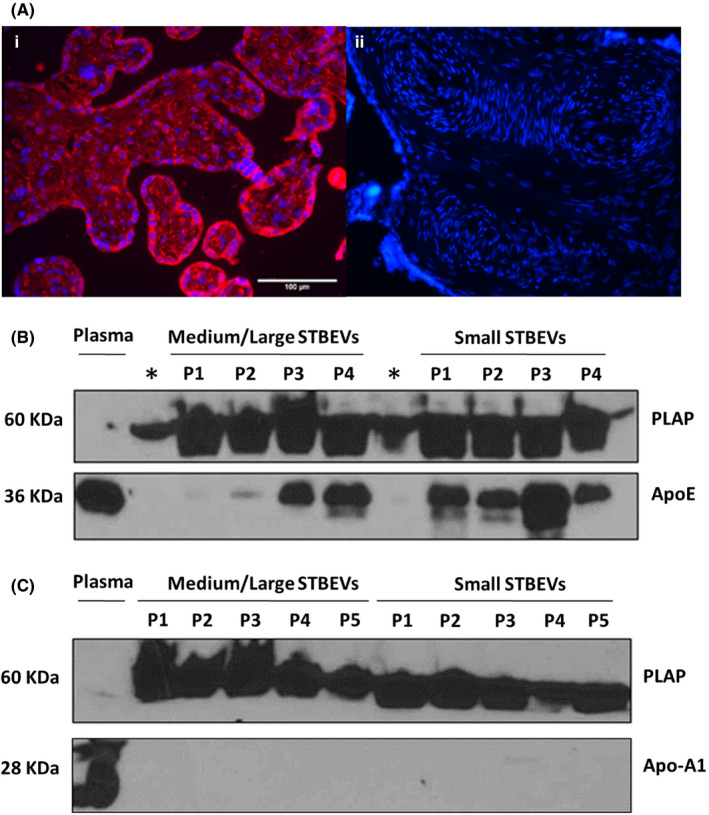
Expression of ApoE in the human placenta and STBEVs derived from normal pregnancy. (A) Representative immunofluorescence analysis demonstrating (i) apoE expression (red) on the syncytiotrophoblast layer of placental chorionic villi. Cell nuclei staining has been performed with DAPI (blue); (ii) negative control with anti‐ IgG rabbit antibody (n = 6). (B) Representative immunoblot demonstrating apoE (36 KDa) and PLAP (60 KDa) expression on medium/large and small STBEVs derived from normal placentae (P1‐4) (n = 4). *Empty wells with sample overflowed from neighbouring wells. (C) Representative immunoblot showing lack of expression of apo‐A1 (28KDa) on the STBEVs derived from normal placentae (P1‐5) (n = 5). Plasma derived from NP patient at term was used as positive control for lipoproteins ApoE and Apo‐A1. P: placental lysate; STBEVs: syncytiotrophoblast‐derived extracellular vesicles; medium/large STBEVs: STBEV isolated at 10,000xg centrifugation; small STBEVs, STBEV isolated at 150,000xg centrifugation; apoE, apolipoprotein‐E; apo‐A1, apolipoprotein A1

Immunofluorescence staining of placental sections displayed high expression of apoE in the syncytiotrophoblast layer (Figure 2A). Western blot analysis demonstrated expression of both PLAP and apoE in plasma, medium/large and small STBEVs **(**Figure 2B). Particularly, apoE expression seemed higher and with less variable concentration in small than in medium/large vesicles. An alternate apolipoprotein apo‐A1 (which was not detected in our proteomics analysis) was detected in plasma but was not detected in either medium/large or small STBEVs confirming that apoE expression in STBEV was specific, and not due to lipoproteins contamination of STBEVs during ultracentrifugation process.

### 3.3 Immunoaffinity depletion confirms that PLAP and apoE are expressed on the same vesicles

**FIGURE 3:**
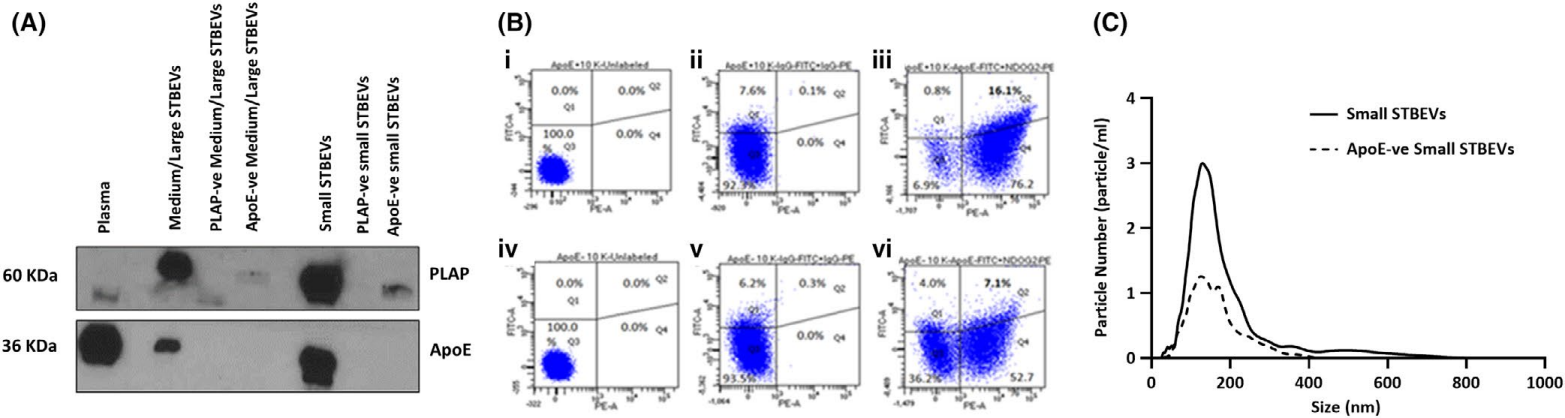
Co‐expression of apoE with PLAP in STBEVs. (A) Representative immunoblot showing PLAP and apoE co‐expression on plasma, medium/large STBEVs pool (total) and medium/large STBEVs supernatant after depletion with PLAP‐ and apoE‐coated Dynabeads (medium/large STBEVs negative for PLAP and apoE); small STBEVs pool (total) and small STBEV supernatant after depletion with PLAP‐ and apoE‐coated Dynabeads (small STBEVs negative for PLAP and apoE). (B) Representative flow cytometry analysis of apoE and PLAP expression in pooled medium/large STBEVs before (i–iii) and after (iv–vi) immunomagnetic depletion for apoE. Unlabelled STBEVs before (i) and after (iv) immunodepletion for apoE, as well as STBEVs labelled with isotype control antibodies FITC‐ or PE‐conjugated, before (ii) and after (v) immunodepletion for apoE, did not show a significant double‐positive population. Medium/large STBEVs labelled with FITC‐conjugated anti‐apoE and PE‐conjugated anti‐PLAP antibodies showed 16±3% of double positivity for apoE and PLAP (iii), that decreased to 7 ± 2% after immune depletion for apoE (vi). (C) Graph showing results from nano tracking analysis (NTA) of pooled small STBEVs before (solid line) and after (dashed line) immune‐magnetic depletion for apoE. ApoE depletion determined a reduction of STBEVs concentration of 55%, suggesting an apoE positivity in small STBEVs of about 45%

Both PLAP and apoE were expressed in pregnant plasma, medium/large and small STBEVs (pooled from placental perfusions) (Figure 3A). After PLAP magnetic bead immunoaffinity depletion, medium/large STBEVs demonstrated complete depletion of both PLAP and apoE. Correspondingly, after apoE magnetic bead immunoaffinity depletion, medium/large STBEVs showed complete depletion of apoE and virtually all PLAP signal. These results suggest that almost all apoE‐positive medium/large STBEVs co‐express PLAP, and vice versa. Small STBEVs showed similar results with no PLAP and no apoE signal when PLAP depletion was applied. ApoE magnetic bead depletion completely denuded the samples of apoE expression, but a small PLAP signal remained, suggesting that there are some PLAP‐positive small STBEVs, which do not express apoE. These data suggest that significant amounts of apoE on STBEV is co‐expressed with PLAP on the same vesicles.

### 3.4 Flow cytometric analysis confirmed co‐expression of apoE with PLAP in medium/large STBEVs

Flow cytometric analysis of medium/large STBEVs showed that 16±3% of vesicles expressed both PLAP (PE+) and apoE (FITC+), further confirming that apoE is co‐expressed with PLAP on STBEVs, not a result of contamination of STBEVs with maternal blood lipoproteins (Figure 3B‐iii). After apoE magnetic immunodepletion, medium/large STBEVs showed a significant decrease of both PLAP‐positive and ApoE‐positive STBEVs (16±3% to 7±2%, reduction of about 6%), corroborating the magnetic immunodepletion findings (Figure 3B‐vi). No double‐positive population for PLAP and apoE was found when incubating STBEVs with medium only without antibodies—negative control (Figure 3B‐i and ‐iv) or in STBEVs labelled with IgG1 isotype control (Figure 3B‐ii and ‐v), demonstrating the specificity of the binding of anti‐PLAP and anti‐HLA‐DR antibodies. Small STBEV cannot be visualized using flow cytometry, since they are below the limit of resolution of this technique, but can be assessed by the use of nanoparticle tracking analysis (NTA).

Using NTA, a modal size of 139±6nm was shown in apoE‐positive small STBEVs, with a similar size distribution (130 ± 4 nm) after depletion for apoE (Figure 3C). Furthermore, after immunodepletion for apoE, we observed a reduction of STBEVs concentration by 55%, thus, indirectly demonstrating that the apoE‐positive small STBEVs comprised 45% of the total, substantially higher than that observed for medium/large STBEVs (about 16%). These data are consistent with the results of apoE obtained by Western blot.

### 3.5 Medium/large and small STBEVs enter HepG2 cells through apoE‐ LDLR interaction

**FIGURE 4:**
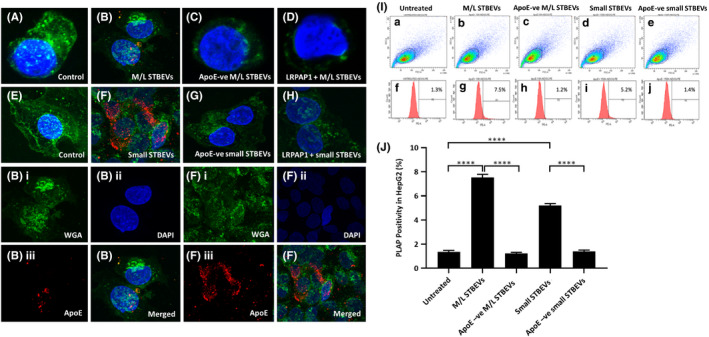
Uptake of STBEVs by HepG2 cells. Panel A‐H: Confocal microscopy of STBEVs treatment to HepG2 cells. Nuclei were detected with DAPI (blue). Cell membranes were labelled with wheat germ agglutinin (WGA) (green). Cells were stained with mouse monoclonal anti‐PLAP antibody (NDOG2) and next with Alexa Fluor^®^ 647 conjugate goat anti‐mouse secondary antibody (red). A, E: untreated cells. HepG2 cells incubated with medium/large STBEVs carrying apoE showed intracellular staining for PLAP (B) that was even higher in cells incubated with small STBEVs (F). No evident staining for PLAP was detected in cells treated with apoE‐depleted medium/large (C) or small (G) STBEVs, or pre‐treated with the LDLR blocker, LRPAP1, and then incubated with medium/large (D) or small (H) STBEVs. Below, split images of B and F, showing separately positive staining for membranes (Bi, Fi), nuclei (Bii, Fii) and PLAP (Biii, Fiii). Panel I: Flow cytometric analysis for PLAP of HepG2 cells. a,f) Untreated HepG2 cells; b,g) HepG2 cells treated with medium/large STBEVs; c,h) apoE‐depleted medium/large STBEVs; d,i) small STBEVs; e‐j) apoE‐depleted small STBEVs. A significant increase of PLAP detection was observed in cells treated with medium/large or small STBEVs (g and i, respectively), compared to untreated HepG cells (f) or cells treated with apoE‐depleted medium/large or small STBEVs (h and j, respectively). Panel J: Results summary graph showing PLAP positivity in HepG2 percentage. Data are expressed as mean of three experiments ±standard deviation. Statistical analysis was performed by using Student's *t*‐test. ****p < 0.0001. M/L: medium/large

Having confirmed that apoE and PLAP were co‐expressed on STBEV, we next assessed uptake of apoE‐containing STBEV by analysing the presence of PLAP in HepG2 cells treated with native and apoE‐depleted vesicles (HepG2 cells do not express PLAP). Confocal microscopy demonstrated uptake of PLAP‐positive medium/large and small STBEVs by HepG2 cells (Figure 4 Panel A–H). Cells incubated with apoE‐depleted STBEVs showed no positivity for PLAP (Figure 4C and G). ApoE usually enters hepatic cells via the LDL receptor.^12^, ^13^ In agreement with this HepG2 cells pre‐treated with LRPAP1 (an LDL‐Receptor inhibitor) showed no positive staining for PLAP either in apoE‐positive vesicles or in ApoE‐depleted vesicles suggesting that LDL Receptor is the principal mode of entry of STBEV into these cells (Figure 4D and H). To confirm and quantify apoE‐positive STBEV uptake by HepG2 cells, flow cytometry analysis was performed (Figure 4 Panel I). HepG2 cells were stained with PE‐conjugated anti‐PLAP antibody. A significantly higher percentage of PLAP‐positive cells was observed in cells treated with pooled medium/large (7.5 ± 1.3%, Figure 4.Ig) or small (5.2 ± 0.8%, Figure 4Ii) STBEVs not depleted for apoE compared to untreated cells (1.3±0.4%, Figure 4.If) or cells incubated with apoE‐depleted medium/large or small STBEVs (1.2±0.3% and 1.4±0.2%, Figure 4.Ih and 4I.j, respectively). These data corroborated our findings that the primary mode of entry of STBEV into HepG2 cells is via ApoE/LDL Receptor.

### 3.6 STBEVs increase synthesis of cholesterol in HepG2 cells

**FIGURE 5:**
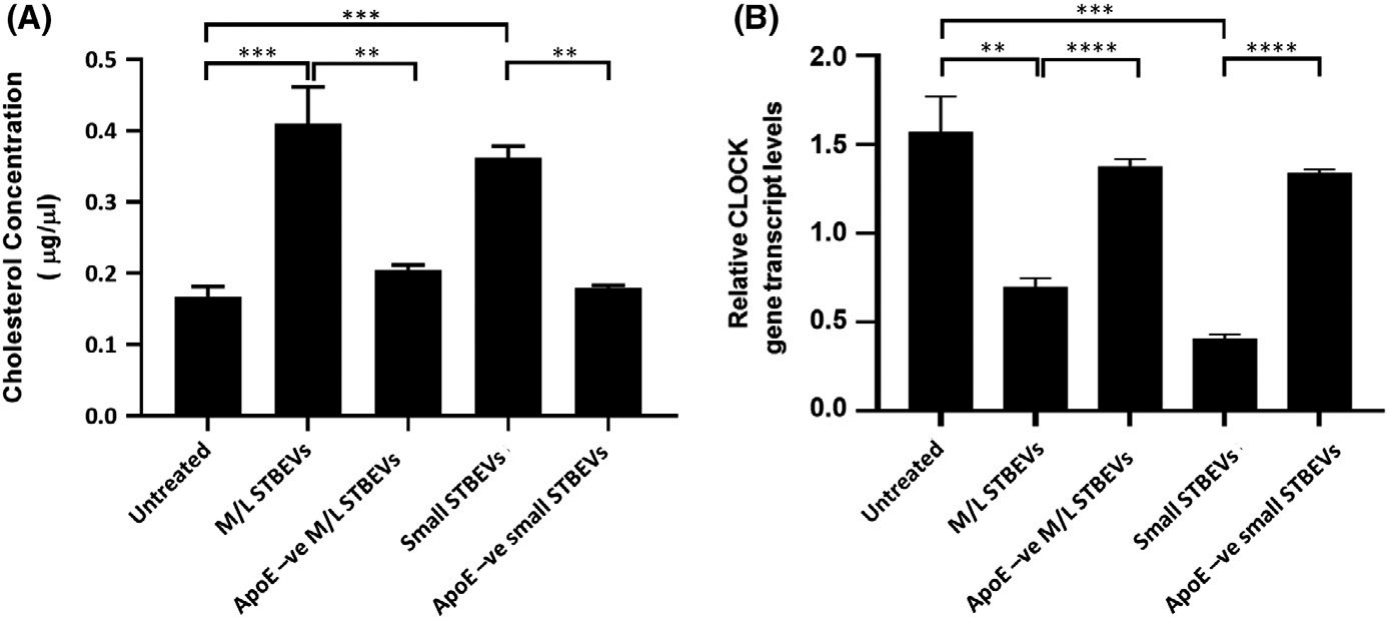
Metabolic effects in HepG2 cells after uptake of apoE‐positive STBEVs. (A) ELISA of cholesterol levels in HepG2 cells lysates. Histograms showing results of cholesterol assays of HepG2 cells lysates after 24 hours of incubation with pooled medium/large or small STBEVs, medium/large or small STBEVs depleted for ApoE (Apo‐ve). Data are expressed as mean of three experiments ±standard deviation. Statistical analysis was performed by using Student's *t*‐test. **p < 0.01;***p < 0.001. (B) CLOCK gene expression in HepG2 assessed by qPCR. After 24 hours of incubation with pooled medium/large or small STBEVs, medium/large or small depleted for apoE (apo –ve), significant reduction of CLOCK gene expression was observed in cells treated with medium/large or small STBEVs. No significant reduction of the gene was found after treatment of HepG2 cells with apoE‐negative medium/large or small STBEVs. Data are expressed as mean of three experiments ±standard deviation. Statistical analysis was performed by using Student's *t*‐test. **p < 0.01; ***p < 0.01; ****p < 0.001

The biological effects of ApoE‐positive STBEV on HepG2 cells was assessed by measurement of cholesterol synthesis. HepG2 cells analysed by ELISA showed significantly higher production of cholesterol when incubated with medium/large or small STBEVs (*p *< 0.001 *versus* untreated) (Figure 5A). When HepG2 cells were treated with either medium/large or small STBEVs depleted of apoE, synthesis of cholesterol did not increase (Figure 5A).

### 3.7 ApoE‐positive STBEVs downregulate CLOCK gene expression in HepG2 cells

We next assessed global changes in the mRNA of HepG2 cells incubated with apoE‐positive STBEV using microarray analysis of 44,000 genes. This revealed a reduced expression of CLOCK (Circadian Locomotor Output Cycles Kaput) gene, a well‐known regulator of circadian cell metabolism. HepG2 cells showed statistically significant decrease in CLOCK gene expression after 24 hours of incubation with either of the apoE‐positive STBEV subpopulations. In particular, apoE‐positive medium/large STBEVs caused 1.17 ‐fold decrease in CLOCK gene expression compared to untreated cells (*p *< 0.01), or to cells incubated with apoE‐negative medium/large STBEVs (0.75‐fold; *p *< 0.0001). This downregulation of CLOCK gene expression was even higher when HepG2 cells were incubated with apoE‐positive small STBEVs compared to untreated cells (2.82 ‐fold; *p *< 0.001) or to cells incubated with apoE‐negative small STBEVs (2.25‐fold; *p *< 0.0001) (Figure 5B). No significant modification of CLOCK gene expression was observed in HepG2 cells treated with apoE‐negative medium/large or small STBEVs. No significant modifications of the other genes analysed were observed in HepG2 cells.

## 4 DISCUSSION

Apolipoprotein‐E is usually carried by chylomicrons and IDL in vivo, where it mediates the hepatic clearance of remnant circulating lipoproteins.^12^ ApoE enters hepatic cells primarily via the LDL receptor and has been documented as being expressed in wide variety of extra‐hepatic tissues.^13^ ApoE secretion by the placenta has been demonstrated^14^ but we are the first to demonstrate that STBEVs from normal term placentae express apoE. This appeared to be specific since interrogation of STBEV for another structurally related apolipoprotein, Apo‐A1 was negative.^15^ Flow cytometry confirmed that apoE was co‐expressed with PLAP—a marker of syncytiotrophoblast. The possibility of EV contamination by lipoproteins during the ultracentrifugation process (used to isolate the STBEVs) was excluded by co‐immunoaffinity precipitation, which showed that STBEVs isolated by PLAP pull‐down were apoE‐positive and vice versa.

Having confirmed that apoE is expressed on STBEVs, we next demonstrated that apoE‐positive STBEVs are taken up by HepG2 cells, a hepatocyte carcinoma line that normally expresses the receptor for low‐density lipoproteins (LDLR), which is a high‐affinity receptor for apoE. LDLR is involved in the interaction with apoE in lipoproteins and mediates a ligand‐receptor binding and consequent uptake of circulating lipoproteins by liver cells in vivo.

HepG2 cells natively express apoE but do not express PLAP. We, therefore, used this feature of the STBEVs (PLAP/ApoE double positivity) to assess the uptake of apoE‐positive STBEVs into HepG2 cells. The apoE‐positive STBEV uptake into HepG2 cells appeared to be the main mode of entry since by depleting vesicles of apoE molecules or blocking the LDLR with human recombinant LRPAP1 (an LDLR inhibitor), we inhibited STBEVs uptake. Use of confocal microscopy and Z‐stack technology allowed us to confirm that PLAP‐positive vesicles were not only bound to HepG2 cell membranes but were actually internalized by cells. This observation has been confirmed and quantified by flow cytometric analysis of HepG2 cells positivity for PLAP, after 24 hours of incubation with STBEVs. Hypothesizing that STBEVs may modulate maternal liver metabolism in normal pregnancy, we investigated the transcriptional effect in liver cells after STBEVs uptake. Gene expression arrays performed on HepG2 cells incubated for 24 hours with apoE‐positive STBEVs demonstrated a significant downregulation of CLOCK gene expression, which was confirmed by qPCR.

The CLOCK gene is a member of the clock gene family, which is central to the cellular regulation of circadian rhythm homeostasis and orchestrates shifts in metabolic patterns to accompany changes in activity and food consumption.^16^, ^17^, ^18^, ^19^, ^20^ Nightly induction of CLOCK gene expression in liver cells reduces glucose and lipid synthesis, corresponding to the reduced energy requirements of diurnal mammals.^16^, ^17^ However, in pregnancy there is significant derangement of lipid synthesis with circadian changes in liver permitting maintenance of nutrient availability in pregnancy.^21^ To confirm a metabolic effect, cholesterol synthesis was investigated in HepG2 cells incubated for 24 hours with apoE‐positive STBEVs and we observed a striking increase in cholesterol synthesis after the uptake of both medium/large and small STBEVs. No significant increase of lipid synthesis was seen after incubation of liver cell with apoE‐negative STBEVs, confirming that apoE expression on STBEVs is required to target liver cells and induce metabolic modifications in pregnancy.

## 5 CONCLUSIONS

Our study provides a novel model of placenta‐liver communication in human pregnancy, mediated by placental extracellular vesicles, that may be, in part, responsible for inducing the circadian‐independent metabolic lipid changes occurring in pregnancy. The results of this study come from in vitro experiments and validation of these observations in vivo is required.

## CONFLICT OF INTEREST

The authors declare that they have no conflicts of interest.

## AUTHOR CONTRIBUTION

**Chiara Tersigni:** Conceptualization (equal); Data curation (equal); Formal analysis (equal); Investigation (equal); Writing‐original draft (equal); Writing‐review & editing (equal). **Muhammad Furqan Bari:** Investigation (supporting); Methodology (supporting); Software (supporting). **Shijie Cai:** Formal analysis (supporting); Investigation (supporting); Validation (supporting). **Wei Zhang:** Investigation (supporting); Methodology (supporting); Writing‐review & editing (supporting). **Neva Kandzija:** Methodology (supporting); Supervision (supporting); Writing‐review & editing (supporting). **Alice Buchan:** Investigation (supporting). **Fabrizio Miranda:** Investigation (equal); Methodology (equal); Software (equal); Writing‐review & editing (equal). **Nicoletta Di Simone:** Methodology (supporting); Resources (equal); Supervision (equal); Writing‐review & editing (equal). **Christopher Redman:** Conceptualization (equal); Supervision (equal); Validation (equal); Writing‐review & editing (equal). **Claire Bastie:** Investigation (equal); Methodology (equal); Supervision (equal); Writing‐review & editing (equal). **Manu Vatish:** Conceptualization (equal); Formal analysis (equal); Funding acquisition (equal); Methodology (equal); Supervision (equal); Writing‐review & editing (equal).

## Supporting information

Fig S1

## Data Availability

The data that support the findings of this study are available from the corresponding author upon reasonable request.
