# Supplementary figures and images for: Syncytiotrophoblast‐derived extracellular vesicles carry apolipoprotein‐E and affect lipid synthesis of liver cells in vitro
Source: J Cell Mol Med. 2021 Dec 10;26(1):123–32. doi: 10.1111/jcmm.17056 (PMC8742183; doi:10.1111/jcmm.17056)

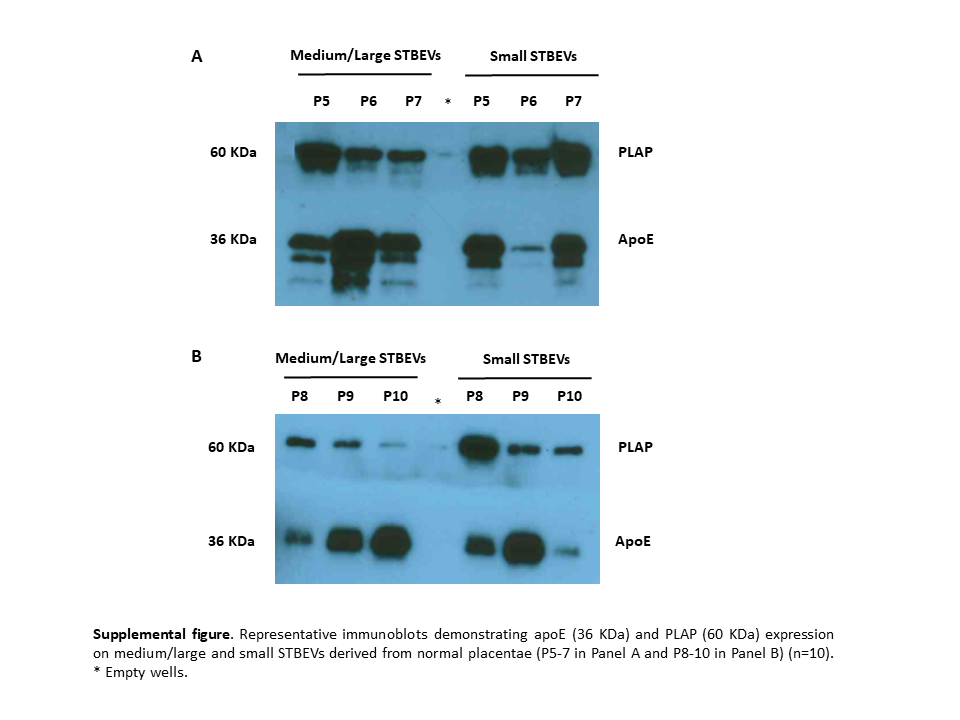

Supplement: Supplementary file 1 — Fig S1 [file JCMM-26-123-s001.tif]
